# *G0S2* Gene Polymorphism and Its Relationship with Carcass Traits in Chicken

**DOI:** 10.3390/ani12070916

**Published:** 2022-04-02

**Authors:** Xin Yang, Yuanrong Xian, Zhenhui Li, Zhijun Wang, Qinghua Nie

**Affiliations:** 1 State Key Laboratory for Conservation and Utilization of Subtropical Agro-Bioresources, Lingnan Guangdong Laboratory of Agriculture, College of Animal Science, South China Agricultural University, Guangzhou 510642, China; yangxin@stu.scau.edu.cn (X.Y.); xianyuanrong97@stu.edu.cn (Y.X.); lizhenhui@scau.edu.cn (Z.L.); zhijunwang@stu.scau.edu.cn (Z.W.); 2 National-Local Joint Engineering Research Center for Livestock Breeding, Guangdong Provincial Key Lab of Agro-Animal Genomics and Molecular Breeding, and Key Laboratory of Chicken Genetics, Breeding and Reproduction, Ministry of Agriculture, Guangzhou 510642, China; 3 College of Animal Science, South China Agricultural University, Guangzhou 510642, China

**Keywords:** *G0S2* gene, single nucleotide polymorphism, carcass traits, association analysis

## Abstract

**Simple Summary:**

With the progress of society, the demand for meat is increasing; therefore, how to improve the efficiency of breeding, reduce environmental pollution, and reduce the cost of breeding have become urgent priorities. Abdominal fat is a redundant part of the production of chickens. Thus, reducing abdominal fat deposition is increasingly vital in chicken breeding. Studies have shown that *G0S2* is a crucial gene in regulating fat metabolism, and its single nucleotide polymorphism (SNP) was significantly associated with chicken production traits in previous studies. The present study aimed to identify SNPs of the *G0S2* gene and analyze whether they were associated with chicken carcass traits, including abdominal fat weight. The present study results can provide practical information for molecular marker-assisted breeding of chicken carcass traits.

**Abstract:**

Gene single nucleotide polymorphisms can be used as auxiliary markers in molecular breeding and are an effective method to improve production performance. *G0S2* is a key gene involved in regulating fat metabolism, but little research has been conducted on this gene regarding its role in poultry. In this study, the specialized commercial partridge chicken strain *G0S2* gene was cloned and sequenced, and the relationship between the SNP sites on *G0S2* and the carcass traits of chickens was investigated. The results showed that a total of seven SNPs were detected on *G0S2* (g.102G > A, g.255G > A, g.349C > T, g.384A > G, g.386G > A, g.444G > A, g.556G > A). Two sites are located in the coding region and five sites are located in the 3′-UTR. SNPs located in the coding region are synonymous mutations. g.444G > A has a significant correlation with abdominal fat weight. The chickens with *AG* and *GG* genotypes have the highest abdominal fat weight, while the *AA* genotype is lower. The g.102G > A genotype has a significant correlation with live and abdominal fat weight. The live weight and abdominal fat weight of the chickens with *AA* and *AG* genotypes are at a higher level and have a larger gap than the *GG* genotype. Chickens with the *AA* genotype in g.556G > A had the lowest fat weight. The results of present study can provide practical information for molecular marker-assisted breeding of chicken carcass traits.

## 1. Introduction

Meat is a good source of protein and energy for human beings. As the global population increases, the demand for meat is also rising [1]. However, at the same time, discussions on the environmental impact of the poultry industry and the efficiency of resource utilization have never stopped. Chicken meat is one of the most consumed meats. At the same time, compared with mammals, chicken production emits less waste. Developed countries such as the United States, the United Kingdom, Canada, and Japan closed live poultry trading markets, and poultry meat circulates in chilled products. In China, consumers prefer to buy live poultry for slaughter. However, the circulation of live poultry may lead to outbreaks such as influenza in birds. It is a general trend for poultry meat to be marketed chilled. Carcass traits are essential traits for chilled whole chicken products that can visually present the product to the consumer. However, carcass traits can only be measured after chicken slaughter, which is challenging to use for breeding production.

Adipose triglyceride lipase (ATGL) is the rate-limiting enzyme that hydrolyzes triacylglycerol in adipocytes. The *G0S2* gene is a regulator of the *ATGL* gene, which can directly bind to *ATGL* to inhibit the activity of *ATGL* and reduce the rate of lipolysis [2,3]. In cattle, researchers have verified that *G0S2* is related to the fat content of cattle by detecting the expression level of *G0S2* in the muscles and different intramuscular fat content in bulls [4]. *G0S2* is highly expressed in pig liver, and to a lesser degree, in omental adipose tissue and suet fatty tissue in pigs. Moreover, two SNPs affect back fat thickness (BFT) [5]. In poultry, the function of *G0S2* is the same as that in mammals. Adipose tissue in the body can act as an inhibitor of ATGL to regulate changes in lipolytic activity [6]. In the meantime, it has been reported that *G0S2* knockout chickens can reduce abdominal fat deposition without affecting other traits, and the fatty acid composition in their blood and abdominal fat is changed [7].

Skeletal muscle is an energy-consuming machine, and fat tissue acts as a buffer pool between energy intake and consumption, and when intake exceeds consumption fat is deposited [8]. *G0S2* has been reported to inhibit *ATGL* activity in mouse and human skeletal muscle and plays an essential role in regulating lipid metabolism and substrate oxidation [9]. In mice, deletion of *G0S2* resulted in a significant reduction in relative body weight gain and a significant increase in serum glycerol levels [10].

Partridge chicken is one of the essential endemic varieties in China, famous for its good flavor [11]. The partridge chicken used in this experiment is a specialized commercial strain of broiler bred by KwangFeng Industrial Co., Ltd. (Guangzhou, China) and South China Agricultural University. It aims to ensure the meat quality and flavor while optimizing the skin color and other properties suitable for the chilled market. The results of this study may provide helpful information for *GOS2* gene marker-assisted selection in chicken production.

## 2. Materials and Methods

### 2.1. Experimental Animals and Determination of Their Carcass Traits

This study took local Chinese flocks of partridge chickens as the research object. All samples and carcass trait data were collected in Guangzhou KwangFeng Industrial Co., Ltd. Guangzhou KwangFeng Industrial Co., Ltd. is the animal experimental unit operated under South China Agricultural University. All animal experiments in this study were conducted in strict accordance with the guidelines of the Guide to Animal Welfare in China. These experiments (approval number: SCAU#2021F074) were carried out under the approval of the College Animal Science, South China Agricultural University (Guangzhou, China). We made every effort to reduce the suffering of animals.

The G genealogy was constructed with 24 male spotted chickens and 86 female yellow chickens as the ancestors; the M3 genealogy was constructed with 76 male spotted chickens and 16 female yellow chickens. This experiment selected 389 F2 generation partridge chickens from G genealogy, and 327 F2 generation partridge chickens were selected from M3 genealogy. Seven hundred sixteen healthy genealogy chickens were used to measure their carcass traits. All chickens were raised in stepped cages to 90 days of age, and they were randomly given foot numbers and slaughtered according to standard procedures. Meanwhile, blood samples were collected and stored at −20 °C. The measured indicators included live weight, dressed weight, half-bore weight, full-bore weight, breast muscle weight, leg weight, wing weight, foot weight, head weight, heart weight, liver weight, stomach weight, abdominal fat weight, shin length, shin circumference (the circumference of the tape measured around the shin), and body oblique length. The measurement of chicken carcass traits and segmentation method referred to Introduction to Animal Husbandry and Chicken carcass segmentation [12,13]. Among them, the breast muscles, legs, wings, feet, shin length, and shin circumference were all measured on the right part of the chicken.

### 2.2. DNA Extraction

We used an E.Z.N.A.^®^ SQ Blood DNA Kit (Omega, Georgia, GA, USA) to extract blood genomic DNA. The concentration of the extracted DNA was measured with a spectrophotometer (Thermo Fisher, Waltham, MA, USA), and the concentration was adjusted to 100–200 ng/μL with double distilled water.

### 2.3. G0S2 Fragment Amplification

We downloaded the *G0S2* full length fragment on NCBI (https://www.ncbi.nlm.nih.gov, accessed on 20 March 2021), and then used NCBI Primer-BLAST (Primer designing tool (https://www.ncbi.nlm.nih.gov/tools/primer-blast/)) to design PCR amplification primers. The total volume of PCR was 30 μL, and the amplification system was 3 uL partridge chicken gDNA at a concentration of 100–200 ng/uL; 1.2 μL upstream primer (Tsingke Biotech, Beijing, China) at a concentration of 10nM; 1.2 μL downstream primer at a concentration of 10 nM; 9.6 μL double distilled water; 15 μL Green Taq Mix (Vazyme, Nanjing, China). The primer sequences of PCR were upstream primer 5′-GCTACACTAACGTGCCCCTC-3′, downstream primer 3′-TTACTGCCCACAGGCGTTC-5′. The PCR program was pre-denatured for the first 5 min, denatured at 95 °C for 15 s, followed by annealing at 58 °C, extension at 72 °C for 50 s (32 cycles), and final extension at 72 °C for 5 min at the end of the cycle. It was stored temporarily at 4 °C until off the machine. Then, the amplified samples were sent to Tsingke Biotech Co., Ltd. for sequencing.

### 2.4. Statistics and Analysis

The sequence peak map file returned by the sequencing was put into Seqman in DNASTAR Lasergene software (DNASTAR, Madison, WI, USA) to open, compared with the downloaded sequence, and the genotype of each SNP was recorded. We used Microsoft Excel (Microsoft Corp., Redmond, WA, USA) to calculate the allele frequency, genotype frequency, Chi-square value, Hardy–Weinberg *p*-value, theoretical heterozygosity, actual heterozygosity, PIC value, and purity of each SNP Degree. Haploview 4.1 was used to analyze the linkage disequilibrium of the collated data [14].

Association analysis of SNPs (or haplotypes) and carcass traits were performed with mixed linear models and were established for each SNP and carcass trait using SPSS 26 software. The mixed linear model used is as follows:

*Y* = *μ* + *G* + *H* + *e*

*Y*: phenotypic values of carcass traits, *μ*: the overall population mean, *G*: the fixed effect of genotype or haplotype, *H*: the fixed effect of hatch, *e*: random residual error. Correction for multiple comparisons was performed using the Bonferroni method.

## 3. Results

### 3.1. SNP and Genotyping of G0S2 Gene

**Figure 1:**
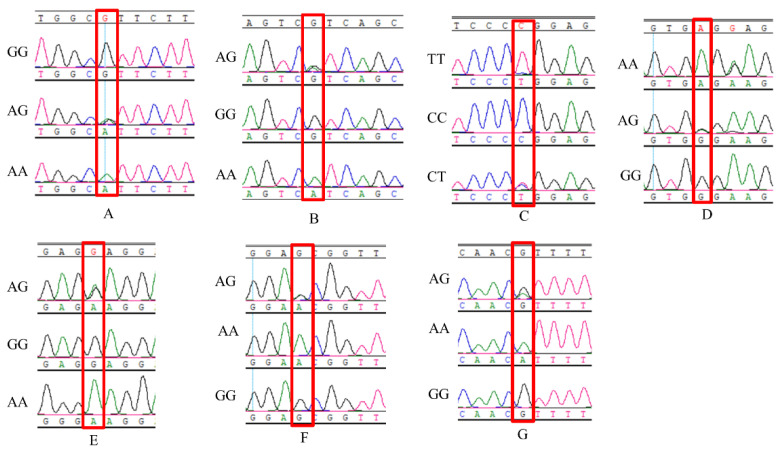
The peak maps of all the three genotypes of SNPs. A: g.102G > A. B: g.255G > A. C: g.349C > T. D: g.384A > G. E: g.386G > A. F: g.444G > A. G: g.556G > A.

PCR was used to amplify fragments of *G0S2*, which contained all coding regions and all fragments of the 3′-UTR. The amplified DNA fragments of 716 individuals were sequenced by second-generation Sanger sequencing, and the base sequence and base peak map of the *G0S2* fragment of 716 individuals were obtained. After comparison and statistics, the sites with genotype frequency greater than 85% and allele frequency less than 1% were removed, and a total of seven SNP sites were screened out on this *G0S2* segment for further research. Among them, two SNP sites are located on exons, and five SNP sites are located on the 3′-UTR. Two SNP sites located on exons did not change the coding nucleotides and belonged to synonymous mutations. We list the position information of the obtained SNP sites on the chromosome in Table 1. The peak maps of all three genotypes of SNPs are shown in Figure 1.

Among the seven SNPs screened, three genotypes were detected by observing the statistical sequencing peak map. We analyzed the statistical results and calculated the genotype frequency and allele frequency of these seven SNPs, as shown in Table 2. We define the allele downloaded from the database as wild type and the mutant allele obtained by sequencing and screening as mutant type. In the five SNPs of g.102G > A, g.255G > A, g.349C > T, g.384A > G, and g.556G > A, the frequency of wild-type alleles is greater than that of mutant alleles. In addition, in the four SNPs of g.102G > A, g.255G > A, g.349C > T, and g.556G > A, the *GG* type (*CC* type) is dominant. In the SNPs of g.384A > G, the distribution of the three genotypes of *GG*, *AG*, and *AA* is relatively close. However, the frequency of wild-type alleles of g.386G > A and g.444G > A is less than that of mutant allele frequency; wild-type allele frequency is only 0.175 and 0.117, respectively. In g.386G > A and g.444G > A, the *AA* genotype is dominant. We also calculated and studied a series of genotype frequency and allele frequency parameters. We performed the Hardy–Weinberg Chi-square test on genotype frequency and allele frequency. The Hardy–Weinberg Chi-square test *p*-value shows that only the SNP of g.386G > A is in Hardy–Weinberg equilibrium. Except for g.386G > A, the genotype frequency and allele frequency of the remaining SNPs have a large gap between the actual observed value and the theoretically inferred value, and the *p*-value is less than 0.05, which is not in the Hardy–Weinberg equilibrium state. We surmise this may be the result of artificial breeding. Among the seven SNPs, g.349C > T, g.386G > A, and g.444G > A are low-level polymorphisms, and g.102G > A, g.255G > A, g.384A > G, and g. 556G > A are moderately polymorphic.

### 3.2. Correlation Analysis of Chicken Carcass Traits

To understand the relationship between various carcass traits in chickens, we calculated the Pearson product-moment correlation coefficient between the traits, as shown in Table 3. The results showed significant correlations among the live weight, dressed weight, half-bore weight, and full-bore weight, and the correlation coefficient was high. In addition to wing and stomach weight, abdominal fat weight was significantly correlated with other carcass traits. However, their correlation coefficients are all at a low level. This phenomenon suggests that abdominal fat needs to be considered an independent aspect in poultry breeding. Although breast muscle weight was significantly correlated with other carcass traits, its correlation coefficient was low. In addition, live weight was significantly correlated with full-bore weight, wing weight, foot weight, shin length, and body oblique length, and had a high correlation coefficient. We speculate that in this strain of chickens, the relationship between chicken body size and meat production rate is lower. The nutrients are used to increase body size, not muscle hypertrophy. In addition, interestingly, there was a significant negative correlation between abdominal fat weight and foot weight, head weight, and shin circumference.

### 3.3. Association Analysis of G0S2 Gene SNPs and Carcass Traits

To study the relationship between the screened SNPs and carcass traits, we performed an association analysis of *GOS2* genotype information with 16 traits. The results of the association analysis are shown in Table 4. The results show that, except for head weight, breast muscle weight, foot weight, and body oblique length, g.102G > A, g.255G > A, g.384A > G, g.444G > A, and g.556G > A, with the remaining 12 traits such as live weight, carcass weight, and abdominal fat weight, are all at significant levels. It is worth noting that the *AA* genotype in g.102G > A has the highest level in these 12 traits. g.349C > T significantly correlates with live weight, dressed weight, leg weight, heart weight, and liver weight. G.386G > A is only significantly related to live weight, heart weight, liver weight, abdominal fat weight, and tibia length. Among these traits, the *AG* genotype is the dominant genotype. It should be noted that, except for g.349C > T, all other SNPs are significant with abdominal fat weight. Another point of importance is that the AG genotype in g.444G > A has the highest abdominal fat weight, and the *AA* genotype in g.556G > A has the lowest abdominal fat weight. Breast muscle weight is only associated with two SNPs, g.444G > A and g.556G > A. Among them, g.444G > A is significantly associated with breast muscle weight, and g.556G > A is significantly associated with breast muscle weight. The AG genotype in g.444G > A has the highest breast muscle weight. In addition, the head weight trait does not correlate with all SNPs.

### 3.4. Linkage Disequilibrium Analysis and Haplotype Analysis of SNPs of G0S2 Gene

**Figure 2:**
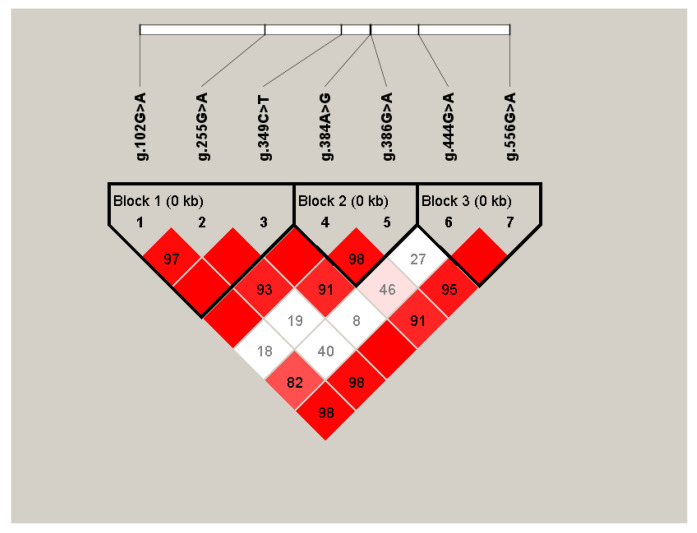
Linkage disequilibrium analysis of SNPs of *G0S2* gene.

The results of linkage disequilibrium analysis for seven SNPs are shown in Figure 2. The LD map shows seven SNPs of the *G0S2* gene, and three vital LD regions were detected.

The analysis results of these three blocks show that block1 has five haplotypes, block2 has four haplotypes, and block3 has three haplotypes. The composition and frequency of the haplotypes are shown in Table 5. In order to study the relationship between these haplotypes and slaughter traits, we conducted a joint analysis. The joint analysis results are shown in Table 6 (we excluded haplotypes with a frequency less than 5%). Our results show that the data of the three haplotype combinations of H1H1, H6H6, and H10H10 are lower than those of other groups, and there are significant differences. We can pay more attention to the production and weed out this type during breeding.

## 4. Discussion

The world’s demand for meat continues to increase, and how to use fewer resources to produce more meat products has become a top priority. Carcass traits are important economic traits of the poultry breeding industry. They can be presented directly to consumers. At the same time, it is also a simple and direct manifestation of the economic benefits of breeding companies. In a pedigree named TT, live weight at 42 days has a high genetic correlation with carcass and carcass part weight but a low genetic correlation with organ weight [15]. Meanwhile, there are also related studies showing that higher body weights and prime cut traits have a higher genetic correlation [16]. Although the pedigrees in the studies mentioned above are different from our study, the conclusions are partially similar and support each other. According to the correlation results between our carcass traits, it is more consistent with the conclusions of the studies mentioned above, that is, the live body weight has higher correlation with half-bore weight, full-bore weight, breast muscle weight, thigh weight, wing weight, and foot weight, but lower correlation with each organ.

Thus far, many studies have found that genome-wide SNP screening, genome-wide haplotype screening, quantitative trait locus (QTL), and SNPs can have a certain impact on the improvement of poultry traits or the improvement of production performance [17,18,19,20,21,22]. However, compared with livestock and other mammals, the improvement of poultry carcass traits started late. Therefore, it is very important to have more research results to enrich the breeding strategy to improve the characteristics of poultry meat carcasses.

In the previous study of our team, we found four SNPs in *G0S2*: g.102G > A, g.111C > T, g.197G > A, and g.255g > A. Association analysis with production traits showed that g.102G > A and g.255G > A were significantly associated with head width and leg muscle color. g.102G > A was significantly associated with shank diameter at 63 days. g.197G > A was significantly associated with shank diameter at 49 days, the crude protein content of leg muscle, and other traits [23]. The two sites, g.102G > A and g.255G > A, are the same as the coding region in this study. However, the traits associated with the two studies were not the same. The previous studies mainly focused on production traits, while this study focused on carcass traits. The selection of carcass traits can be more directly reflected in the slaughtered chickens, which fitted with the trend of unified slaughtering and chilled for sale. Compared with previous studies that only detected SNPs in coding regions, this study detected five SNPs in non-coding regions. SNPs in non-coding regions also significantly affect transcriptional efficiency, gene expression, and splicing dysregulation [24,25].

Through previous studies, *G0S2* is considered one of the key regulatory genes in fat metabolism [24,26]. Studies have shown that *G0S2* directly binds to *ATGL* to reduce the hydrolysis of fat [25]. In the previous study of our team, there were similar results. Knockdown of *G0S2* can activate *ATGL*, thereby promoting triacylglycerol hydrolase activity. At the same time, it affects the *FABP4* gene, thereby inhibiting *PPARγ* and further reducing adipogenesis [27]. Poultry’s abdominal fat is currently considered a by-product of no economic value. Excessive deposition of abdominal fat will affect the economic benefits of production. Abdominal fat deposition and muscle weight gain are related [28,29,30]. Therefore, we believe that the use of *G0S2*, a key gene in fat metabolism, as a molecular marker for carcass traits, assists in improving poultry carcass traits. It has a certain meaning in production. Although some laboratories have been able to breed *G0S2* knockout chickens, the breeding process usually takes two years or more. It takes a long approach to knock out genes from the laboratory to production and use, and the use of molecular markers for selection is a better choice.

In summary, in this study, partial fragments of poultry *G0S2* gene were amplified by PCR, and seven SNPs were obtained (g.102G > A, g.255G > A, g.349C > T, g.384G > A, g.386G> A, g.444G > A, g.556G > A). In addition, the relationship between the *G0S2* gene polymorphism and 16 carcass traits was analyzed. Association analysis found that g.444G > A has a significant correlation with abdominal fat weight. The *AG* and *GG* genotype have the highest abdominal fat weight, while the *AA* genotype is lower. The g.102G > A genotype has a significant correlation with live and abdominal fat weight. The live weight and abdominal fat weight of the *AA* and *AG* genotypes are at a higher level and have a larger gap than the *GG* genotype. The *AA* genotypes of g.102G > A, g.255G > A, and g.384G > A are significantly different from the *GG* genotype, and the *AA* genotype had significantly higher values of the traits than the *GG* genotype. However, g.386G > A, g.444G > A, and g.556G > A have opposite trends. In addition, the three haplotypes of H1H1, H6H6, and H10H10 are also worthy of our attention in production. Therefore, this study finds that the seven SNPs, g.102G > A, g.255G > A, g.349C > T, g.384G > A, g.386G > A, g.444G > A, and g.556G > A, can be used as additional molecular markers for carcass improvement, providing a theoretical basis for poultry breeding.

## 5. Conclusions

In summary, this study detected seven SNPs of the *G0S2* gene and analyzed the relationship between their polymorphisms and chicken carcass traits. At the same time, the correlation between each carcass trait was investigated. The results indicated that abdominal fat weight and breast muscle weight were significantly correlated with other carcass traits, but the correlation coefficients were all at low levels. Chickens with AA genotype in g.102G > A had the highest level among 12 traits, except for head weight, breast muscle weight, foot weight, and body oblique length. Chickens with the AG genotype in g.444G > A had the highest abdominal fat weight, while chickens with the AA genotype in g.556G > A had the lowest. These SNPs can be used for marker-assisted selection to improve carcass traits in partridge chickens. This will be helpful in breeding programs aimed at improving chicken carcass traits.

## Figures and Tables

**Table 1:** SNPs detected on partial fragments of *G0S2*.

SNPs	Chromosome Position	Location	Amino Acid Change
g.102G > A	3176848	Exon	Synonymous mutation
g.255G > A	3177001	Exon	Synonymous mutation
g.349C > T	3177095	3′-UTR	Untranslated point mutation
g.384A > G	3177130	3′-UTR	Untranslated point mutation
g.386G > A	3177132	3′-UTR	Untranslated point mutation
g.444G > A	3177190	3′-UTR	Untranslated point mutation
g.556G > A	3177302	3′-UTR	Untranslated point mutation

**Table 2:** The genotype frequency, allele frequency and diversity parameters of *G0S2*.

Gene	SNPs	Genotype Frequency			Allelic Frequency		*p*-Value ^1^	Ho	He	PIC ^2^	Ne
*G0S2*		GG	AG	AA	G	A					
	g.102G > A	0.630	0.281	0.089	0.770	0.230	0.000	0.281	0.354	0.291	1.390
	g.255G > A	0.613	0.274	0.113	0.750	0.250	0.000	0.274	0.375	0.305	1.377
	g.384A > G	0.385	0.380	0.235	0.575	0.425	0.000	0.380	0.489	0.369	1.613
	g.386G > A	0.029	0.292	0.679	0.175	0.825	0.967	0.292	0.289	0.247	1.412
	g.444G > A	0.047	0.140	0.813	0.117	0.883	0.000	0.140	0.207	0.186	1.162
	g.556G > A	0.447	0.366	0.187	0.630	0.370	0.000	0.366	0.466	0.358	1.577
		CC	CT	TT	C	T					
	g.349C > T	0.941	0.049	0.010	0.964	0.034	0.000	0.049	0.069	0.067	1.051

Abbreviations: SNPs = single nucleotide polymorphism sites; *p*-Value: the χ^2^ test of Hardy–Weinberg equilibrium; Ho: observed heterozygosity; He: expected heterozygosity; PIC: polymorphism information content; Ne: effective number of alleles. ^1^ *p*-value > 0.05 means it is in Hardy Weinberg equilibrium. ^2^ PIC > 0.5 means high polymorphism, 0.25 < PIC < 0.5 means medium polymorphism, PIC < 0.25 means low polymorphism.

**Table 3:** Correlation coefficients (r) between carcass traits.

	LW	DW	HBW	FBW	BMW	TW	WW	FW	HW	HEW	LIW	SW	AFW	SL	SC	BOL
LW	1															
DW	0.973 **	1														
HBW	0.977 **	0.970 **	1													
FBW	0.973 **	0.968 **	0.985 **	1												
BMW	0.363 **	0.357 **	0.362 **	0.373 **	1											
TW	0.909 **	0.910 **	0.926 **	0.933 **	0.326 **	1										
WW	0.889 **	0.885 **	0.900 **	0.908 **	0.315 **	0.867 **	1									
FW	0.834 **	0.847 **	0.851 **	0.848 **	0.234 **	0.853 **	0.833 **	1								
HW	0.720 **	0.743 **	0.755 **	0.749 **	0.208 **	0.760 **	0.740 **	0.849 **	1							
HEW	0.737 **	0.716 **	0.721 **	0.701 **	0.206 **	0.646 **	0.642 **	0.626 **	0.538 **	1						
LIW	0.695 **	0.675 **	0.660 **	0.630 **	0.193 **	0.579 **	0.588 **	0.562 **	0.449 **	0.601 **	1					
SW	0.472 **	0.456 **	0.422 **	0.395 **	0.122 **	0.367 **	0.385 **	0.408 **	0.325 **	0.352 **	0.354 **	1				
AFW	0.263 **	0.219 **	0.238 **	0.198 **	0.161 **	0.110 **	0.06	−0.115 **	−0.186 **	0.227 **	0.168 **	−0.005	1			
SL	0.831 **	0.793 **	0.803 **	0.797 **	0.274 **	0.730 **	0.731 **	0.674 **	0.509 **	0.656 **	0.619 **	0.376 **	0.290 **	1		
SC	0.452 **	0.490 **	0.493 **	0.501 **	0.089 *	0.536 **	0.529 **	0.722 **	0.697 **	0.320 **	0.252 **	0.190 **	−0.344 **	0.263 **	1	
BOL	0.774 **	0.771 **	0.775 **	0.778 **	0.300 **	0.755 **	0.712 **	0.686 **	0.559 **	0.535 **	0.499 **	0.381 **	0.183 **	0.668 **	0.370 **	1

Abbreviations: LW: live weight (g); DW: dressed Weight (g); HBW: half-bore weight (g); FBW: full-bore weight (g); BMW: breast muscle weight (g); TW: thigh weight (g); WW: wing weight (g); FW: foot weight (g); HW: head heavy (g); HEW: heart weight (g); LIW: liver weight (g); SW: stomach weight (g); AFW: abdominal fat weight (g); SL: shin length (mm); SC: shin circumference (cm); BOL: body oblique length (cm). ** Indicates a significant correlation at the level of 0.001 (2-tailed), and * indicates a significant correlation at the level of 0.05 (2-tailed).

**Table 4:** Association analysis of *G0S2* SNPs with carcass traits (MEAN ± SEM).

Genotypes	SNP	AA/TT (MEAN ± SEM)	AG/CT (MEAN ± SEM)	GG/CC (MEAN ± SEM)
LW	g.102G > A	1782.42 + 173.25 ^A^	1710.32 + 202.16 ^A^	1597.43 + 224.22 ^B^
	g.255G > A	1762.28 + 161.52 ^A^	1716.50 + 205.67 ^A^	1592.51 + 224.69 ^B^
	g.384A > G	1721.36 + 196.17 ^A^	1672.64 + 214.79 ^A^	1572.98 + 227.45 ^B^
	g.386G > A	1628.14 + 224.32 ^a^	1681.26 + 219.27 ^b^	1696.66 + 212.00 ^ab^
	g.444G > A	1621.14 + 226.80 ^a^	1755.30 + 181.44 ^b^	1742.94 + 151.23 ^b^
	g.556G > A	1557.91 + 227.15 ^A^	1588.35 + 226.97 ^A^	1729.32 + 188.50 ^B^
	g.349C > T	1672.85 + 115.57 ^ab^	1768.28 + 190.60 ^b^	1639.01 + 224.43 ^a^
DW	g.102G > A	1566.95 + 157.32 ^A^	1503.19 + 180.07 ^A^	1412.25 + 197.23 ^B^
	g.255G > A	1547.52 + 147.36 ^A^	1509.11 + 183.44 ^B^	1408.24 + 197.69 ^B^
	g.384A > G	1511.77 + 174.53 ^A^	1472.31 + 190.71 ^A^	1394.58 + 200.63 ^B^
	g.386G > A	1439.13 + 197.96	1476.33 + 192.06	1494.26 + 188.95
	g.444G > A	1432.59 + 199.14 ^a^	1535.56 + 170.41 ^b^	1530.25 + 134.11 ^b^
	g.556G > A	1388.19 + 201.80 ^A^	1403.61 + 201.09 ^A^	1517.46 + 169.22 ^B^
	g.349C > T	1464.57 + 93.83 ^ab^	1544.42 + 176.23 ^b^	1446.65 + 197.33 ^a^
HBW	g.102G > A	1417.60 + 145.15 ^a^	1355.63 + 162.44 ^b^	1274.83 + 180.57 ^c^
	g.255G > A	1400.72 + 135.24 ^A^	1360.23 + 165.90 ^B^	1271.28 + 180.97 ^B^
	g.384A > G	1364.07 + 159.06 ^A^	1330.03 + 173.06 ^A^	1258.06 + 183.98 ^B^
	g.386G > A	1299.42 + 180.55	1332.31 + 176.19	1342.23 + 165.58
	g.444G > A	1292.47 + 181.88 ^a^	1386.47 + 151.39 ^b^	1390.95 + 125.50 ^b^
	g.556G > A	1248.63 + 184.01 ^A^	1268.03 + 184.54 ^A^	1370.68 + 152.91 ^B^
	g.349C > T	1316.28 + 80.88 ^ab^	1400.89 + 162.22 ^b^	11305.51 + 179.81 ^a^
FBW	g.102G > A	1171.10 + 123.91 ^a^	1120.60 + 135.91 ^b^	1055.40 + 149.83 ^c^
	g.255G > A	1157.34 + 115.17 ^A^	1124.75 + 139.07 ^B^	1052.35 + 149.99 ^B^
	g.384A > G	1129.26 + 134.46 ^A^	1099.20 + 144.10 ^A^	1041.60 + 151.80 ^B^
	g.386G > A	1074.61 + 149.28	1103.46 + 148.24	1109.13 + 136.47
	g.444G > A	1070.32 + 151.56 ^a^	1141.46 + 125.43 ^b^	1150.17 + 109.59 ^b^
	g.556G > A	1034.45 + 149.81 ^A^	1050.00 + 153.88 ^A^	1132.70 + 129.28 ^B^
	g.349C > T	1081.88 + 70.32 ^ab^	1158.89 + 133.36 ^b^	1080.18 + 149.52 ^a^
BMW	g.102G > A	89.10 + 10.47	86.86 + 12.17	83.55 + 31.54
	g.255G > A	88.84 + 9.94	87.17 + 12.46	83.28 + 31.89
	g.384A > G	86.37 + 11.44	86.14 + 12.52	82.98 + 39.12
	g.386G > A	84.78 + 30.50	85.21 + 12.75	87.13 + 9.69
	g.444G > A	83.13 + 12.56 ^a^	94.22 + 62.08 ^b^	89.39 + 8.98 ^ab^
	g.556G > A	79.29 + 12.33 ^a^	85.11 + 40.17 ^ab^	87.24 + 11.17 ^b^
	g.349C > T	84.20 + 7.71	90.11 + 12.36	84.72 + 26.72
TW	g.102G > A	210.70 + 28.00 ^a^	200.20 + 29.81 ^b^	190.20 + 29.95 ^c^
	g.255G > A	207.08 + 26.22 ^A^	200.96 + 30.48 ^A^	189.85 + 30.06 ^B^
	g.384A > G	202.54 + 27.39 ^A^	196.79 + 30.81 ^A^	188.22 + 30.55 ^B^
	g.386G > A	193.31 + 30.64	197.85 + 30.05	200.18 + 27.88
	g.444G > A	187.54 + 31.09 ^A^	188.94 + 30.84 ^A^	202.72 + 27.97 ^B^
	g.556G > A	187.54 + 31.09 ^A^	188.94 + 30.84 ^A^	202.72 + 27.97 ^B^
	g.349C > T	191.19 + 7.88 ^ab^	209.79 + 28.71 ^b^	194.10 + 30.49 ^a^
WW	g.102G > A	68.90 + 9.37 ^A^	66.22 + 8.05 ^A^	62.64 + 9.14 ^B^
	g.255G > A	67.99 + 8.66 ^A^	66.54 + 8.53 ^A^	62.47 + 9.02 ^B^
	g.384A > G	66.79 + 8.69 ^A^	65.00 + 8.71 ^A^	61.86 + 9.23 ^B^
	g.386G > A	63.66 + 9.06	65.24 + 9.28	66.66 + 7.84
	g.444G > A	63.50 + 9.22 ^a^	67.20 + 8.01 ^b^	67.47 + 8.02 ^b^
	g.556G > A	61.48 + 9.40 ^A^	62.30 + 8.98 ^A^	66.91 + 8.37 ^B^
	g.349C > T	61.59 + 4.37	67.63 + 8.88	64.06 + 9.13
FW	g.102G > A	75.84 + 14.98 ^a^	72.77 + 14.03 ^a^	69.82 + 13.94 ^b^
	g.255G > A	74.10 + 13.83 ^a^	73.49 + 14.65 ^a^	69.62 + 13.83 ^b^
	g.384A > G	73.058 + 13.96 ^a^	71.68 + 13.84 ^ab^	69.56 + 14.51 ^b^
	g.386G > A	70.80 + 14.15	72.03 + 14.24	71.73 + 14.51
	g.444G > A	70.64 + 14.31	73.93 + 13.94	72.40 + 11.73
	g.556G > A	69.89 + 14.87 ^a^	69.10 + 13.97 ^a^	73.43 + 13.76 ^b^
	g.349C > T	65.37 + 7.73	74.20 + 14.97	71.09 + 14.17
HW	g.102G > A	50.10 + 8.63	49.14 + 9.09	48.21 + 9.84
	g.255G > A	49.19 + 8.23	49.55 + 9.31	48.13 + 9.84
	g.384A > G	48.70 + 8.44	48.73 + 9.51	48.51 + 10.20
	g.386G > A	48.66 + 9.71	48.83 + 9.32	46.37 + 7.37
	g.444G > A	48.46 + 9.78	49.19 + 8.46	50.05 + 8.39
	g.556G > A	49.64 + 11.38	47.38 + 9.31	49.25 + 8.77
	g.349C > T	43.89 + 2.80	49.04 + 9.38	48.67 + 9.59
HEW	g.102G > A	8.37 + 1.83 ^A^	7.87 + 1.84 ^A^	7.09 + 1.81 ^B^
	g.255G > A	8.39 + 1.75 ^A^	7.85 + 1.83 ^A^	7.06 + 1.81 ^B^
	g.384A > G	8.05 + 1.82 ^A^	7.57 + 1.82 ^A^	6.90 + 1.82 ^B^
	g.386G > A	7.29 + 1.88 ^a^	7.70 + 1.83 ^b^	7.71 + 1.95 ^ab^
	g.444G > A	7.21 + 1.86 ^a^	8.35 + 1.72 ^b^	8.35 + 1.43 ^b^
	g.556G > A	6.69 + 1.76 ^A^	7.00 + 1.80 ^A^	8.08 + 1.77 ^B^
	g.349C > T	7.53 + 1.18 ^ab^	8.35 + 1.74 ^b^	7.37 + 1.88 ^a^
LIW	g.102G > A	35.22 + 4.51 ^A^	34.31 + 5.94	31.51 + 6.26 ^B^
	g.255G > A	34.70 + 4.33 ^A^	34.43 + 6.04 ^A^	31.44 + 6.29 ^B^
	g.384A > G	34.38 + 5.88 ^A^	33.38 + 6.18 ^A^	30.82 + 5.98 ^B^
	g.386G > A	32.15 + 6.00 ^a^	33.59 + 6.51 ^b^	34.11 + 6.71 ^ab^
	g.444G > A	32.13 + 6.26 ^a^	34.72 + 5.65 ^b^	35.10 + 4.88 ^b^
	g.556G > A	30.43 + 5.79 ^A^	31.22 + 6.21 ^A^	34.70 + 5.73 ^B^
	g.349C > T	33.51 + 8.49 ^ab^	35.84 + 6.82 ^b^	32.45 + 6.11 ^a^
SW	g.102G > A	32.65 + 5.77 ^a^	32.30 + 5.91 ^a^	30.38 + 6.05 ^b^
	g.255G > A	32.61 + 5.83 ^a^	32.26 + 6.03 ^a^	30.34 + 6.00 ^b^
	g.384A > G	30.98 + 5.89 ^a^	31.46 + 6.38 ^a^	31.38 + 6.43 ^b^
	g.386G > A	30.92 + 5.91	31.57 + 6.36	31.38 + 6.44
	g.444G > A	30.69 + 5.86 ^a^	33.27 + 7.00 ^b^	32.13 + 5.04 ^b^
	g.556G > A	29.81 + 5.62	30.26 + 5.61	32.38 + 6.36
	g.349C > T	32.49 + 5.50	33.41 + 7.98	30.99 + 5.93
AFW	g.102G > A	45.14 + 13.74 ^A^	41.39 + 14.62 ^A^	35.13 + 15.45 ^B^
	g.255G > A	45.18 + 13.53 ^A^	41.58 + 14.50 ^A^	34.72 + 15.43 ^B^
	g.384A > G	43.05 + 14.24 ^A^	39.12 + 15.10 ^A^	33.25 + 15.36 ^B^
	g.386G > A	36.68 + 15.60 ^a^	40.02 + 14.84 ^b^	40.95 + 16.89 ^ab^
	g.444G > A	35.81 + 15.16 ^A^	46.34 + 14.74 ^B^	46.25 + 11.52 ^B^
	g.556G > A	31.33 + 13.78 ^A^	34.55 + 15.85 ^A^	43.12 + 14.07 ^B^
	g.349C > T	49.19 + 15.81	43.57 + 14.59	37.36 + 15.44
SL	g.102G > A	73.65 + 5.51 ^A^	72.12 + 6.38 ^A^	68.53 + 7.06 ^B^
	g.255G > A	73.48 + 5.08 ^A^	72.29 + 6.48 ^A^	68.33 + 7.04 ^B^
	g.384A > G	72.46 + 6.11 ^A^	71.01 + 6.87 ^A^	67.50 + 6.92 ^B^
	g.386G > A	69.33 + 6.94 ^a^	71.37 + 7.06 ^b^	71.81 + 6.33 ^ab^
	g.444G > A	69.00 + 7.01 ^A^	74.25 + 5.55 ^B^	74.48 + 4.12 ^B^
	g.556G > A	66.25 + 6.53 ^a^	68.09 + 7.02 ^a^	73.12 + 5.79 ^c^
	g.349C > T	72.70 + 3.97 ^ab^	74.10 + 5.39 ^b^	69.75 + 7.05 ^a^
SC	g.102G > A	11.18 + 1.22	11.10 + 0.98	11.28 + 1.06
	g.255G > A	11.03 + 1.13	11.15 + 1.00	11.25 + 1.06
	g.384A > G	11.08 + 1.03	11.17 + 1.07	11.29 + 1.04
	g.386G > A	11.19 + 1.03	11.24 + 1.09	10.97 + 1.15
	g.444G > A	11.23 + 1.03	11.07 + 1.16	10.91 + 1.05
	g.556G > A	11.40 + 1.07 ^a^	11.20 + 0.99 ^ab^	11.11 + 1.08 ^b^
	g.349C > T	10.24 + 0.61 ^b^	10.98 + 0.99 ^ab^	11.22 + 1.05 ^a^
BOL	g.102G > A	20.05 + 1.10 ^A^	19.74 + 1.09 ^A^	19.30 + 1.16 ^B^
	g.255G > A	19.95 + 1.04 ^A^	19.76 + 1.12 ^A^	19.29 + 1.16 ^B^
	g.384A > G	19.84 + 1.12 ^A^	19.62 + 1.10 ^A^	19.15 + 1.17 ^B^
	g.386G > A	19.39 + 1.16	19.69 + 1.14	19.71 + 1.10
	g.444G > A	19.40 + 1.18	19.92 + 1.01	19.79 + 0.89
	g.556G > A	19.14 + 1.19 ^A^	19.25 + 1.16 ^A^	19.83 + 1.06 ^B^
	g.349C > T	19.40 + 0.72 ^ab^	20.23 + 1.01 ^b^	19.45 + 1.16 ^a^

Abbreviations: LW: live weight (g); DW: dressed weight (g); HBW: half-bore weight (g); FBW: full-bore weight (g); BMW: breast muscle weight (g); TW: thigh weight (g); WW: wing weight (g); FW: foot weight (g). HW: head heavy (g); HEW: heart weight (g); LIW: liver weight (g); SW: stomach weight (g); AFW: abdominal fat weight (g); SL: shin length (mm); SC: shin circumference (cm); BOL: body oblique length (cm). Different lowercase letters indicate significant differences in means (*p* < 0.05), different capital letters indicate highly significant differences in means (*p* < 0.001), and the same letters indicate insignificant differences in means (*p* > 0.05).

**Table 5:** Haplotype composition of the three linkage regions.

Block	Haplotype	SNPs			Haplotype Frequency
		g.102G > A	g.255G > A	g.349C > T	
Block1	H1	G	G	C	0.712
	H2	G	G	T	0.034
	H3	G	A	C	0.024
	H4	A	G	C	0.003
	H5	A	A	C	0.226
		g.384A > G	g.386G > A		
Block2	H6	G	A		0.574
	H7	G	G		0.001
	H8	A	A		0.251
	H9	A	G		0.174
		g.444G > A	g.556G > A		
Block3	H10	A	A		0.370
	H11	A	G		0.513
	H12	G	G		0.117

**Table 6:** Association analysis of haplotypes of G0S2 SNPs with carcass traits (MEAN ± SEM).

Carcass Traits	Haplotype	MEAN ± SEM	Haplotype	MEAN ± SEM	Haplotype	MEAN ± SEM
LW	H1H1	1582.65 + 225.36 ^A^	H6H6	1572.15 ± 227.92 ^A^	H10H10	1557.91 ± 227.15 ^A^
	H1H5	1709.21 ± 207.92 ^B^	H6H8	1688.99 ± 205.91 ^B^	H10H11	1576.54 ± 227.56 ^A^
	H5H5	1782.42 ± 173.25 ^B^	H6H9	1652.84 ± 224.32 ^B^	H11H11	1712.67 ± 195.34 ^B^
			H8H8	1727.77 ± 175.65 ^B^	H11H12	1768.5 ± 179.91 ^B^
			H8H9	1722.73 ± 208.24 ^B^		
DW	H1H1	1401.00 ± 198.86 ^A^	H6H6	1393.97 ± 201.02 ^A^	H10H10	1388.19 ± 201.80 ^A^
	H1H5	1503.14 ± 185.41 ^B^	H6H8	1488.67 ± 186.27 ^B^	H10H11	1395.35 ± 202.87 ^A^
	H5H5	1566.96 ± 157.32 ^B^	H6H9	1452.5 ± 194.87 ^AB^	H11H11	1503.63 ± 172.36 ^B^
			H8H8	1518.41 ± 156.51 ^B^	H11H12	1549.35 ± 171.59 ^B^
			H8H9	1511.17 ± 185.13 ^B^		
HBW	H1H1	1264.33 ± 182.17 ^A^	H6H6	1257.5 ± 184.38 ^A^	H10H10	1248.63 ± 184.01 ^A^
	H1H5	1354.50 ± 167.24 ^B^	H6H8	1344.55 ± 168.17 ^B^	H10H11	1260.46 ± 186.17 ^A^
	H5H5	1417.61 ± 145.15 ^B^	H6H9	1312.45 ± 177.90 ^AB^	H11H11	1357.18 ± 156.43 ^B^
			H8H8	1374.99 ± 139.01 ^B^	H11H12	1398.37 ± 151.25 ^B^
			H8H9	1361.35 ± 172.17 ^B^		
FBW	H1H1	1046.59 ± 151.07 ^A^	H6H6	1041.16 ± 152.11 ^A^	H10H10	1034.45 ± 149.81 ^A^
	H1H5	1119.86 ± 140.32 ^B^	H6H8	1110.51 ± 140.42 ^B^	H10H11	1044.91 ± 155.86 ^A^
	H5H5	1171.10 ± 123.918 ^B^	H6H9	1085.5 ± 147.87 ^AB^	H11H11	1122.38 ± 133.12 ^B^
			H8H8	1135.21 ± 116.67 ^B^	H11H12	1152.99 ± 124.85 ^B^
			H8H9	1129.82 ± 146.95 ^B^		
BMW	H1H1	82.9 ± 33.08	H6H6	83.01 ± 39.26	H10H10	79.29 ± 12.33
	H1H5	86.69 ± 12.33	H6H8	87.09 ± 12.46	H10H11	82.43 ± 12.67
	H5H5	89.1 ± 10.47	H6H9	84.98 ± 12.56	H11H11	86.38 ± 11.80
			H8H8	86.99 ± 9.40	H11H12	88.63 ± 10.08
			H8H9	85.71 ± 13.19		
TW	H1H1	188.89 ± 30.22 ^A^	H6H6	188.19 ± 30.61 ^A^	H10H10	187.54 ± 31.09 ^A^
	H1H5	200.12 ± 30.69 ^B^	H6H8	198.36 ± 30.97 ^B^	H10H11	187.96 ± 31.23 ^A^
	H5H5	210.7 ± 28.00 ^B^	H6H9	194.89 ± 30.63 ^AB^	H11H11	201.22 ± 27.32 ^B^
			H8H8	203.64 ± 25.34 ^B^	H11H12	205.44 ± 30.94 ^B^
			H8H9	202.31 ± 28.99 ^B^		
WW	H1H1	62.28 ± 9.11 ^A^	H6H6	61.83 ± 9.22 ^A^	H10H10	61.48 ± 9.40 ^A^
	H1H5	66.26 ± 8.23 ^B^	H6H8	65.76 ± 8.18 ^B^	H10H11	62.08 ± 9.07 ^A^
	H5H5	68.90 ± 9.37 ^B^	H6H9	64.08 ± 9.26 ^AB^	H11H11	66.42 ± 8.56 ^B^
			H8H8	66.61 ± 8.57 ^B^	H11H12	67.99 ± 7.97 ^B^
			H8H9	66.95 ± 9.07 ^B^		
FW	H1H1	69.62 ± 13.98	H6H6	69.54 ± 14.52	H10H10	69.89 ± 14.87 ^AB^
	H1H5	73.18 ± 14.44	H6H8	72.41 ± 13.84	H10H11	69.07 ± 14.17 ^A^
	H5H5	75.84 ± 14.98	H6H9	70.79 ± 13.84	H11H11	72.92 ± 13.87 ^AB^
			H8H8	72.47 ± 12.87	H11H12	75.29 ± 14.26 ^B^
			H8H9	73.82 ± 14.71		
HW	H1H1	48.25 ± 10.02	H6H6	48.45 ± 10.18	H10H10	49.64 ± 11.38
	H1H5	49.43 ± 9.27	H6H8	48.87 ± 9.43	H10H11	47.39 ± 9.53
	H5H5	50.1 ± 8.63	H6H9	48.56 ± 9.66	H11H11	48.93 ± 8.82
			H8H8	49.04 ± 8.35	H11H12	49.76 ± 8.86
			H8H9	49.03 ± 8.74		
HEW	H1H1	6.99 ± 1.81 ^A^	H6H6	6.88 ± 1.82 ^A^	H10H10	6.69 ± 1.76 ^A^
	H1H5	7.78 ± 1.81 ^B^	H6H8	7.7 ± 1.80 ^B^	H10H11	6.9 ± 1.77 ^A^
	H5H5	8.37 ± 1.83 ^B^	H6H9	7.41 ± 1.83 ^AB^	H11H11	7.91 ± 1.81 ^B^
			H8H8	8.11 ± 1.85 ^B^	H11H12	8.43 ± 1.74 ^B^
			H8H9	8.09 ± 1.78 ^B^		
LIW	H1H1	31.17 ± 6.17 ^A^	H6H6	30.82 ± 6.00 ^A^	H10H10	30.43 ± 5.79 ^A^
	H1H5	34.29 ± 5.98 ^B^	H6H8	33.66 ± 5.87 ^B^	H10H11	31.17 ± 6.33 ^A^
	H5H5	35.22 ± 4.51 ^B^	H6H9	33.04 ± 6.54 ^B^	H11H11	34.3 ± 5.89 ^B^
			H8H8	34.35 ± 4.76 ^B^	H11H12	35.6 ± 5.59 ^B^
			H8H9	34.48 ± 6.47 ^B^		
SW	H1H1	30.09 ± 5.74 ^A^	H6H6	29.97 ± 5.69 ^A^	H10H10	29.81 ± 5.62 ^A^
	H1H5	32.10 ± 6.04 ^B^	H6H8	31.71 ± 5.78 ^AB^	H10H11	29.97 ± 5.61 ^A^
	H5H5	32.65 ± 5.77 ^AB^	H6H9	31.28 ± 6.73 ^AB^	H11H11	32.09 ± 6.06 ^B^
			H8H8	33.16 ± 6.36 ^B^	H11H12	33.29 ± 7.57 ^B^
			H8H9	31.96 ± 5.81 ^AB^		
AFW	H1H1	34.05 ± 15.32 ^A^	H6H6	33.27 ± 15.30 ^A^	H10H10	31.33 ± 13.78 ^A^
	H1H5	41.00 ± 14.63 ^B^	H6H8	39.76 ± 14.53 ^B^	H10H11	32.7 ± 14.64 ^A^
	H5H5	45.14 ± 13.74 ^B^	H6H9	38.34 ± 15.79 ^AB^	H11H11	42.25 ± 14.47 ^B^
			H8H8	44.19 ± 15.40 ^B^	H11H12	44.12 ± 13.87 ^B^
			H8H9	42.73 ± 12.64 ^B^		
SL	H1H1	67.96 ± 7.00 ^A^	H6H6	67.47 ± 6.93 ^A^	H10H10	66.25 ± 6.53 ^A^
	H1H5	72.1 ± 6.47 ^B^	H6H8	71.41 ± 6.49 ^B^	H10H11	67.78 ± 7.13 ^A^
	H5H5	73.65 ± 5.51 ^B^	H6H9	70.52 ± 7.30 ^B^	H11H11	72.16 ± 5.92 ^B^
			H8H8	72.46 ± 5.37 ^B^	H11H12	75.12 ± 5.50 ^B^
			H8H9	72.62 ± 6.61 ^B^		
SC	H1H1	11.3 ± 1.05	H6H6	11.29 ± 1.04	H10H10	11.4 ± 1.07
	H1H5	11.14 ± 1.01	H6H8	11.11 ± 1.01	H10H11	11.23 ± 0.99
	H5H5	11.18 ± 1.22	H6H9	11.24 ± 1.13	H11H11	11.13 ± 1.03
			H8H8	10.92 ± 0.99	H11H12	11.13 ± 1.23
			H8H9	11.22 ± 1.03		
BOL	H1H1	19.24 ± 1.15 ^A^	H6H6	19.15 ± 1.17 ^A^	H10H10	19.14 ± 1.192 ^A^
	H1H5	19.73 ± 1.10 ^B^	H6H8	19.68 ± 1.07 ^B^	H10H11	19.22 ± 1.19 ^A^
	H5H5	20.05 ± 1.10 ^B^	H6H9	19.55 ± 1.12 ^B^	H11H11	19.77 ± 1.08 ^B^
			H8H8	19.79 ± 1.10 ^B^	H11H12	20.01 ± 1.06 ^B^
			H8H9	19.90 ± 1.15 ^B^		

Abbreviations: LW: live weight (g); DW: dressed weight (g); HBW: half-bore weight (g); FBW: full-bore weight (g); BMW: breast muscle weight (g); TW: thigh weight (g); WW: wing weight (g); FW: foot weight (g). HW: head heavy (g); HEW: heart weight (g); LIW: liver weight (g); SW: stomach weight (g); AFW: abdominal fat weight (g); SL: shin length (mm); SC: shin circumference (cm); BOL: body oblique length (cm). Different lowercase letters indicate significant differences in means (*p* < 0.05), different capital letters indicate highly significant differences in means (*p* < 0.001), and the same letters indicate insignificant differences in means (*p* > 0.05).

## Data Availability

The data presented in this study are available on request from the corresponding author.
